# *Hericium coralloides* Ameliorates Alzheimer’s Disease Pathologies and Cognitive Disorders by Activating Nrf2 Signaling and Regulating Gut Microbiota

**DOI:** 10.3390/nu15173799

**Published:** 2023-08-30

**Authors:** Yue Guan, Dongyu Shi, Shimiao Wang, Yueying Sun, Wanyu Song, Shuyan Liu, Chunyue Wang

**Affiliations:** 1 Engineering Research Center of Chinese Ministry of Education for Edible and Medicinal Fungi, School of Plant Protection, Jilin Agricultural University, Changchun 130118, China; guanyue@mails.jlau.edu.cn (Y.G.); wangshimiao@mails.jlau.edu.cn (S.W.); 2 College of Plant Protection, Jilin Agricultural University, Changchun 130118, China; 2106100121@mails.jlau.edu.cn (D.S.); sunyueying0727@163.com (Y.S.); wanyusongyj@163.com (W.S.)

**Keywords:** Alzheimer’s disease, *Hericium coralloides*, oxidative stress, gut microbiota, nuclear factor erythroid 2-related factor 2

## Abstract

Alzheimer’s disease (AD) is prone to onset and progression under oxidative stress conditions. *Hericium coralloides* (HC) is an edible medicinal fungus that contains various nutrients and possesses antioxidant properties. In the present study, the nutritional composition and neuroprotective effects of HC on APP/PS1 mice were examined. Behavioral experiments showed that HC improved cognitive dysfunction in APP/PS1 mice. Immunohistochemical and Western blotting results showed that HC reduced the levels of p-tau and amyloid-β deposition in the brain. By altering the composition of the gut microbiota, HC promoted the growth of short-chain fatty acid-producing bacteria and suppressed the growth of *Helicobacter*. Metabolomic results showed that HC decreased D-glutamic acid and oxidized glutathione levels. In addition, HC reduced the levels of reactive oxygen species, enhanced the secretion of superoxide dismutase, catalase, and glutathione peroxidase, inhibited the production of malondialdehyde and 4-hydroxynonenal, and activated the nuclear factor erythroid 2-related factor 2 (Nrf2) signaling pathway. Collectively, HC demonstrated antioxidant activity by activating Nrf2 signaling and regulating gut microbiota, further exerting neuroprotective effects. This study confirms that HC has the potential to be a clinically effective AD therapeutic agent and offers a theoretical justification for both the development and use of this fungus.

## 1. Introduction

Alzheimer’s disease (AD) is a neurodegenerative disorder characterized by cognitive dysfunction, which usually presents with memory loss [1]. The World Alzheimer Report 2021 estimates the global prevalence to be approximately 50 million people, with the number of people affected rising to 139 million by 2050 [2]. The primary pathological characteristics of AD include extracellular senile plaques with amyloid-β (Aβ) peptide aggregation and intracellular neurofibrillary tangles with excessive hyperphosphorylation of tau protein [3,4]. In recent years, hypotheses about Alzheimer’s disease mainly include the Aβ cascade hypothesis, tau protein hypothesis, cholinergic injury hypothesis, metal ion disorder hypothesis, oxidative stress hypothesis, and neuroinflammation theory, but there is no theory that can reasonably and comprehensively explain its pathogenesis [5,6,7].

Although the pathogenesis of Alzheimer’s disease remains unclear, there is increasing evidence that gut microbes, through their own metabolites or exogenous substances (prebiotics, natural products, etc.), regulate their composition in the gut, thereby playing a neuroprotective role by alleviating oxidative stress [8]. In an AD mouse model induced by Aβ_42_ injection into the hippocampus, probiotics (*Lactobacillus acidophilus*, *Bifidobacterium lactobacillus*, etc.) reduced oxidative stress and improved their cognitive impairment by reducing malondialdehyde (MDA) content [9]. Probiotics (*B. animalis*) significantly increase the levels of superoxide dismutase (SOD), catalase (CAT), and glutathione peroxidase (GSH-Px) in the plasma and liver of D-galactose-induced oxidative stress model rats and significantly decrease MDA levels [10]. Short-chain fatty acids (SCFAs) are major metabolites of the gut microbiota that play a defensive role in central nervous system diseases by regulating oxidative stress and blood–brain barrier integrity [11]. Supplementation with SCFAs decreased MDA levels and oxidative damage and alleviated cognitive impairment and Aβ accumulation in the brains of AD mice [12]. A key regulator of oxidative stress is nuclear factor erythroid 2-related factor 2 (Nrf2). The pathway linked to Nrf2 is considered a critical antioxidant signaling pathway [13]. Nrf2 eventually binds to antioxidant response elements, triggering phase II gene expression—heme oxygenase-1 (HO-1) and NAD(P)H: quinine oxidoreductase (NQO1), among others [14]—thereby inhibiting oxidative stress, reducing Aβ deposition and tau phosphorylation, and exerting neuroprotection [15].

Edible and medicinal mushrooms have great antioxidant potential owing to their various active ingredients and are excellent sources of natural antioxidants [16]. *Hericium erinaceus*, China’s famous rare and valuable edible mushroom, is an important component of a wide range of food and pharmaceutical products [17] and has shown therapeutic potential in hyperlipidemia [18], neurodegenerative diseases [19], and cancer [20]. Previous studies have shown that the extraction of *H. erinaceus* increases antioxidant levels, improves mitochondrial membrane potential, inhibits apoptosis, and plays a neuroprotective role in H_2_O_2_-induced HT22 cells [21]. In addition, our group confirmed that the mycelial polysaccharide of *H. erinaceus* alleviated oxidative stress and inhibited Ca^2+^ overload in APP/PS1 mice by regulating Nrf2 and its downstream kinases [22]. *Hericium coralloides* (HC) belongs to the *Hericium* genus, Hericiaceae family, and Russulales order with *H. erinaceus* [23]. Extracellular polysaccharides of HC exhibit anticancer activity by inhibiting AGS and MKN-45 cells, based on strong 1,1-diphenyl-2-picrylhydrazyl radical scavenging activity [24]. In D-galactose-induced aging mice, the HC extract significantly improved SOD, CAT, and GSH-Px levels and decreased MDA levels [25]. However, no studies to date have reported the effects of HC on AD.

In this study, we detected the main components of HC, explored the neuroprotective effect of HC on APP/PS1 mice through behavioral experiments, immunohistochemistry, gut microbiota, metabolomics, detection of biochemical indicators, immunofluorescence, and Western blotting, and clarified its mechanism.

## 2. Materials and Methods

### 2.1. HC Preparation

HC fruiting bodies were obtained by artificial cultivation (strain 2020071401 was provided and identified by the Engineering Research Center of the Chinese Ministry of Education for Edible and Medicinal Fungi of Jilin Agricultural University). The composition of the culture substrate was 60–65% moisture, 85% sawdust, 10% wheat bran, 3% soybean meal, 1% maize flour, and 1% gypsum. The substrate was sterilized in an autoclave at 121 °C for 0.5 h after being placed in heat resistant polypropylene bags (15 cm × 37 cm). After cooling to room temperature, the sterilized substrate was inoculated with 30 mL of the liquid culture strain. After mycelial colonization of the substrate, the bags were exposed to a temperature of 18 °C in a controlled chamber to stimulate mushroom production. During mycelial growth and primordium initiation, the relative humidity of the room was altered to 60–70% and 70–85%, respectively. The fruiting bodies were dried via pulverization using a grinding stirrer.

### 2.2. Nutritional Component, Mineral, and Heavy Metal Analyses

The nutrient components of HC were systematically analyzed according to our previous research methods [26,27]. The total ash content was evaluated by weighing the residue recovered after 24 h of burning at 550 °C. The Kjeldahl technique was used to calculate crude protein content [28]. Crude fat content was measured using Soxhlet extraction with petroleum ether as the solvent. The phenol–sulfuric acid technique was used to determine total sugar content [29]. The contents of reducing sugar and crude fiber were detected using the 3,5-dinitrosalicylic acid colorimetric [30] and Ankom filter bag methods [31], respectively. High-performance liquid chromatography was used to measure mannitol content [32]. The levels of total alkaloids [33], triterpenoids [34], sterol [35], flavonoids, and saponins [36] were determined using UV spectrophotometry.

An appropriate amount of the sample was added to a Teflon digestion tank and 5 mL of nitric acid was added. After standing and completion of reaction, the sample was placed in a microwave digestion apparatus. After digestion at 100 °C, 140 °C, 160 °C, and 180 °C for 3 min each and 190 °C for 15 min, ICP-MS was used to detect the following minerals and heavy metal ions: potassium (K), magnesium (Mg), calcium (Ca), sodium (Na), zinc (Zn), iron (Fe), manganese (Mn), selenium (Se), copper (Cu), chromium (Cr), cadmium (Cd), arsenic (As), lead (Pb), and mercury (Hg).

### 2.3. Animals and Ethical Statement

**Figure 1:**
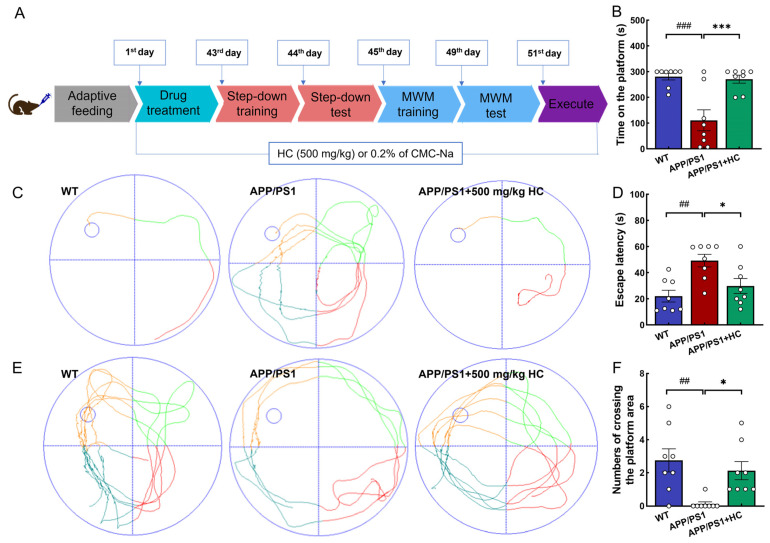
HC treatment improved cognitive and behavioral disorders in APP/PS1 mice. (**A**) Schematic diagram of mouse experiment process. (**B**) HC treatment prolonged the residence time of APP/PS1 mice on the platform in the step-down test. (**C**) Representative trajectories of MWM navigation test. (**D**) HC decreased the escape latency of APP/PS1 mice in the MWM navigation test. (**E**) Representative trajectories of the MWM probe trial (no platform). (**F**) HC treatment increased the crossing times of APP/PS1 mice in the MWM probe trial (no platform). The data are expressed as mean ± S.E.M. (*n* = 8). ^##^ *p* < 0.01, ^###^ *p* < 0.001 vs. WT mice; * *p* < 0.05, *** *p* < 0.001 vs. APP/PS1 mice.

All animal experiments were performed in accordance with the Institutional Animal Ethics Committee of Jilin Agricultural University (Permit Number: 20220615001). Age-matched wild-type (WT) male mice and B6C3-Tg (APPswePSEN1dE9)/Nju double transgenic male mice (APP/PS1) (genotype: (Appswe) T, (Psen1) T) were purchased from Liaoning Changsheng Biotechnology Co., Ltd. (Liaoning, China). Prior to the experiment, the animals were acclimatized to the new environment for 1 week and maintained at a 12 h light/dark cycle at 22 ± 2 °C and 50 ± 10% humidity. Food and water were provided ad libitum. After a week of acclimatization, WT mice were designated as the control group (*n* = 8, 0.2% CMC-Na, 5 mL/kg). The APP/PS1 mice were randomly divided into two groups: APP/PS1 group (*n* = 8, 0.2% sodium carboxymethyl cellulose (CMC-Na), 5 mL/kg), 500 mg/kg HC-treated (APP/PS1+HC) group (*n* = 8, dissolved in 0.2% of CMC-Na, 5 mL/kg). For 49 d, medication was administered intragastrically to all mice at the same time every day (9:00 a.m.). The behavioral experiments began after 42 d of treatment. They were designed to assess learning and memory-related parameters, including a step-down test and Morris water maze (MWM). Finally, the mice were euthanized and blood and tissues were immediately collected for further analysis (Figure 1A).

### 2.4. Behavioral Tests

#### 2.4.1. Step-Down Test

The short-term memory of the mice was examined using a step-down test [22]. The step-down apparatus (SDT-8, Chengdu Techman Software Co., Ltd., Chengdu, China) consisted of an experimental box (658 mm × 395 mm × 450 mm) and a platform (45 mm × 45 mm); the mice were placed in the experimental box for 60 s. Following the adaptation period, they were electroshocked (36 V continuous for 300 s). Under normal conditions, the mice jumped onto the platform when electroshocked. The experiment officially began 24 h after the first training session, and the mice were placed on the platform to record the time spent. Any latency greater than 300 s was counted as 300 s.

#### 2.4.2. MWM Test

The MWM test was used to evaluate the learning and memory abilities of APP/PS1 mice. Similar to previous research [37], the experimental mice underwent 4 d of training before the formal test, 4 times a day (at least once from the other side of the platform; the other three quadrants were randomly placed); the navigation experiment and the probe experiment started on days 49 and 50, and the mice were placed in a circular pool filled with titanium dioxide (SA201, Jiangsu SANS Software Co., Ltd., Jiangsu, China). On day 49, mice were placed in the pool from the other side of the platform and the time taken to locate the platform was measured. On day 50, after the platform was removed, the number of crossed platform areas was recorded.

### 2.5. Hematoylin and Eosin (H&E) and Immunohistochemistry Staining

In accordance with our previous research [38], the collected tissue samples (heart, liver, spleen, kidney, and brain) were fixed in 4% paraformaldehyde solution, dehydrated in 50–100% ethanol and distilled water, then paraffin embedded, cut into 5 μm slices, dewaxed twice in xylene, twice in 100–75% anhydrous ethanol, and finally hydrated. All specimens were stained with H&E. The pathological status of the collected tissue samples was analyzed, sections were observed, and images were collected using a standing optical microscope (Eclipse E100, Nikon, Tokyo, Japan).

As in our previous study [39], after dewaxing and hydration, the brain sections were added to a citric acid antigen retrieval buffer (pH 6.0) (G1202, Servicebio, Beijing, China) for antigen repair. Tissues were evenly covered with 3% bovine serum albumin and incubated at room temperature for 30 min. The tissues were then left overnight along with antibody Aβ_1–42_ (bs-0107R, Bioss, Beijing, China). The phosphate buffer solution (PBS) was washed thrice before incubation with horseradish peroxidase-labeled goat anti-rabbit antibody (E-AB-1003, Elabscience, Wuhan, China) at 25 °C for 1 h. The sections were then restrained with diaminobenzidine tetrachloride solution and hematoxylin and observed under a microscope (Eclipse E100).

### 2.6. Gut Microbiota Analysis

As reported in our previous study [27], the cecal contents of the three groups of mice (WT, APP/PS1, and HC-treated APP/PS1) were evaluated. The 16S rRNA gene was amplified using near-full-length polymerase chain reaction (forward primer 27F: 5′-AGAGTTTGATCMTGGCTCAG-3′ and reverse primer 1492R: 5′-ACCTTGTTACGACTT-3′), DNA extraction, DNA quality determination, and high throughput sequencing for 16S rRNA analysis.

### 2.7. Non-Targeted Metabolomics Analysis of Serum Samples

A pre-cooled methanol/acetonitrile/aqueous solution (2:2:1, *v*/*v*) was pre-chilled, and 100 μL of mouse serum was added. The mixture was stirred by eddy current and ultrasound at low temperature for 30 min, held at −20 °C degrees for 10 min, and centrifuged at 14,000× *g* for 20 min at 4 °C; the supernatant was then vacuum dried. In the mass spectrometer, 100 μL of acetonitrile solution (acetonitrile:water = 1:1, *v/v*) was added for redissolution, eddy current, centrifugation at 4 °C for 15 min. Ultra-high-performance liquid chromatography (UHPLC, 1290 Infinity LC; Agilent Technologies, Santa Clara, CA, USA) and triple quadrupole mass spectrometry (AB SCIEX TripleTOF 6600; AB SCIEX, Framingham, MA, USA) were used to analyze the supernatant and separate the metabolites under the same conditions as previously reported [40].

The total difference in the identified metabolites was assessed using orthogonal partial least squares discriminant analysis (OPLS-DA). Correlations between metabolites with significant differences were analyzed based on correlation analysis methods (*p* < 0.05). Statistical significance was determined by one-way analysis of variance followed by Dunnett’s multiple comparison test, and the associated metabolic pathways were evaluated using the Kyoto Encyclopedia of Genes and Genomes (KEGG).

### 2.8. Determination of Biochemical Indexes

The three groups of brain tissue samples collected were homogenized in normal saline using a high-throughput tissue grinder (Scientz-48, Scientz, Ningbo, China) and centrifuged twice at 4 °C using a centrifuge (3K15, Sigma, Neustadt, Germany). A Pierce bicinchoninic acid (BCA) protein assay kit (23225, Thermo Fisher Scientific, Carlsbad, CA, USA) was used to measure the protein concentration. Kits (Nanjing Jiancheng Biotechnology Co., Ltd., Nanjing, China) were used to measure the GSH-Px (A005-1-2), CAT (A007-1-1), SOD (A001-1-1), and MDA contents (A003-1-1). Reactive oxygen species (ROS) (MM-43700M1) levels in the serum and brain samples were assessed using enzyme-linked immunosorbent assay kits from Jiangsu Enzymatic Immunity Biotechnology Co., Ltd. (Shenzhen, China).

### 2.9. Immunofluorescence

Slides containing brain slices were washed in PBS (pH 7.4) thrice for 5 min each, sealed with 3% bovine serum albumin (BSA) for 30 min, and incubated at 4 °C overnight with 4-hydroxynonenal (4-HNE) (bs-6313R, Bioss, Shanghai, China) and Nrf2 (A1244, ABclonal, Wuhan, China). After washing with PBS, the secondary antibody (GB21404; Servicebio, Wuhan, China) was incubated for 50 min in the dark before imaging with a positive fluorescence microscope (NIKON ECLIPSE C1, Nikon). Semi-quantitative analysis of the images was performed using Image Pro Plus (version 6.0; Media Cybernetics, Rockville, MD, USA).

### 2.10. Western Blotting

Mouse brain tissues were lysed with a 1% protease and phosphatase inhibitor mixture in radioimmunoprecipitation assay buffer (PC 101, Epizyme, Shanghai, China) to extract total protein. The total protein concentration in the supernatant was determined using a BCA protein assay kit. The supernatant was denatured by heating in a metal bath for 5 min. Sodium dodecyl sulfate-polyacrylamide gel electrophoresis separated 45 μg of protein, which was subsequently transferred to a polyvinylidene fluoride membrane (0.45 μm) (10600023, Cytiva, Breisgau, Germany). The membrane was incubated with the primary antibody at 4 °C overnight after being sealed with 5% BSA for 5 h (Table S1). After repeated washing, the membrane was incubated with a suitable enzyme-labeled secondary antibody (Table S1) at 4 °C for 4 h. Finally, an ultra-high-sensitivity enhanced chemiluminescence kit (GK10008, GLPBIO, Montclair, NJ, USA) reagent was used for imprinting. Densitometric analysis of the protein bands was performed using ImageJ software (version 6.0; National Institutes of Health, Bethesda, MD, USA).

### 2.11. Statistical Analysis

Data are expressed as mean ± standard error of the mean (S.E.M.) and calculated using GraphPad Prism 9.3.1 (GraphPad Software Inc., San Diego, CA, USA) with Brown–Forsythe and Bartlett’s one-way analysis of variance followed by Tukey’s post-hoc test. Statistical significance was set at *p* < 0.05.

## 3. Results

### 3.1. The Main Composition of HC

Our results showed that the HC content was 33.40% total sugar, 16.00% crude protein, 11.70% total ash, 7.95% reducing sugar, 6.30% crude fat, 4.60% crude fiber, 3.32% total triterpenoids, 1.10% total saponins, 0.88% total flavonoids, and 0.57% total alkaloids. The total sterol and phenol contents were 0.43% and 0.18%, respectively. The total sugar content was the highest. Among the eight minerals detected in the HC, K content was the highest, and the concentrations of the six heavy metals were low (Table 1).

### 3.2. Effects of HC Treatment on Cognitive Impairments in APP/PS1 Mice

The body weights of the mice in the three groups varied within the normal range during the experiment (Figure S1A). There were no significant differences in the indexes of the hearts (Figure S1B), livers (Figure S1C), spleens (Figure S1D), and kidneys (Figure S1E) of WT, APP/PS1, and APP/PS1+HC mice, and no significant pathological changes were observed in the H&E staining results (Figure S1F–J), indicating the safety of HC administration.

In the step-down test, APP/PS1 mice spent significantly less time on the platform than WT mice (*p* < 0.001). In contrast, HC-treated APP/PS1 mice spent a significantly longer time on the platform (*p* < 0.001) (Figure 1B). The MWM test was used to evaluate the learning and memory abilities of the mice in each group. In the navigation test, APP/PS1 mice had a longer escape latency than WT mice (*p* < 0.01), indicating that APP/PS1 mice exhibited cognitive and spatial memory loss. However, HC treatment dramatically decreased the escape latency in APP/PS1 mice (*p* < 0.05) (Figure 1C,D). In the probe trial, HC treatment significantly increased the number of crossings of the platform area (*p* < 0.05) (Figure 1E,F). These results indicate that HC improved AD-like symptoms in APP/PS1 mice.

### 3.3. HC Treatment Alleviated Aβ and p-tau Deposition in APP/PS1 Mice

**Figure 2:**
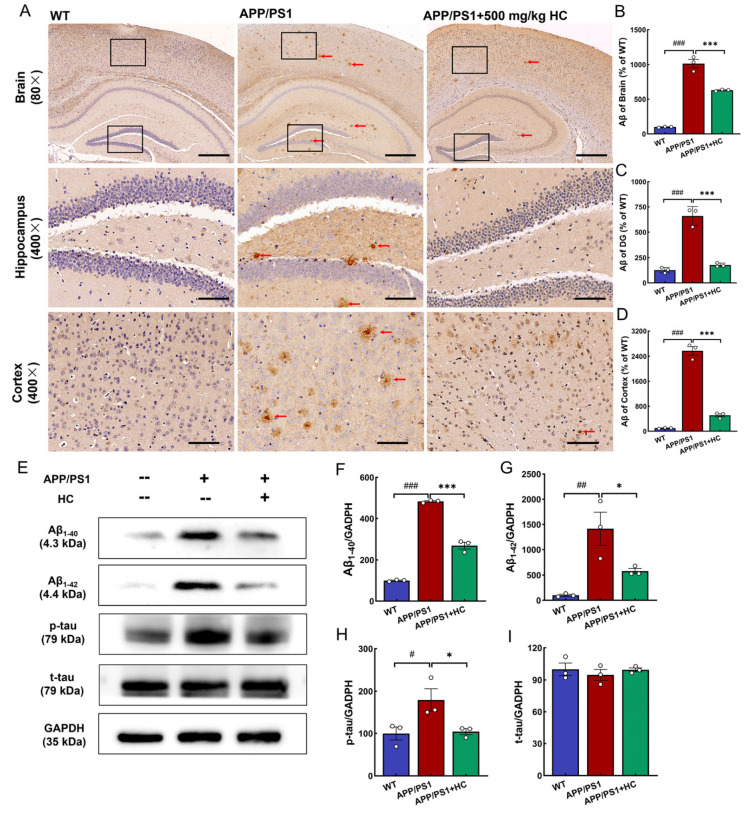
HC reduced the accumulation of Aβ and p-tau in the brains of APP/PS1 mice. (**A**) HC reduced deposition of Aβ (red arrow). Scale bar = 100 μm for 80× magnification and 20 μm for 400× magnification. Quantification of the mean density of Aβ in the (**B**) brain, (**C**) hippocampus, and (**D**) cortex of APP/PS1 mice. (**E**) HC decreased the expression of Aβ_1-40_, Aβ_1-42_, and p-tau in the brains of APP/PS1 mice. Normalized to the levels of glyceraldehyde-3-phosphate dehydrogenase (GAPDH) and quantification of the levels of (**F**) Aβ_1-40_, (**G**) Aβ_1-42_, (**H**) p-tau, and (**I**) t-tau expressed as percentage of the corresponding WT mice. Data are expressed as mean ± S.E.M. (*n* = 3). ^#^ *p* < 0.05, ^##^ *p* < 0.01, ^###^ *p* < 0.001 vs. WT mice; * *p* < 0.05, *** *p* < 0.001 vs. APP/PS1 mice.

APP/PS1 mice are commonly used as an AD model. These mice co-expressed amyloid precursor protein and presenilin-1, showing plaque aggregation and significant cognitive impairment at 8 months [41]. Aβ_1-40_ and Aβ_1-42_ are produced via the amyloidogenic pathway [42,43]. Excessive production or aggregation of Aβ leads to inflammatory cascade reactions, neurofibrillary tangles, axon damage, and synaptic loss, and then causes neuronal apoptosis [44], aggravating the process of AD. Compared to APP/PS1 mice, HC improved the pathological status and significantly inhibited the Aβ plaque area in the hippocampus and cortex (*p* < 0.001) (Figure 2A–D). Western blotting results showed that Aβ_1-40_ and Aβ_1-42_ were downregulated in the brains of APP/PS1 mice after HC treatment (*p* < 0.05) (Figure 2E–G). Additionally, p-tau was significantly reduced by HC treatment in APP/PS1 mice, with no significant difference observed in t-tau, suggesting that HC reduced tau hyperphosphorylation (*p* < 0.05) (Figure 2E,H,I). These results suggested that HC reduced Aβ and p-tau deposition in APP/PS1 mice.

### 3.4. HC Regulated the Gut Microbiota in APP/PS1 Mice

**Figure 3:**
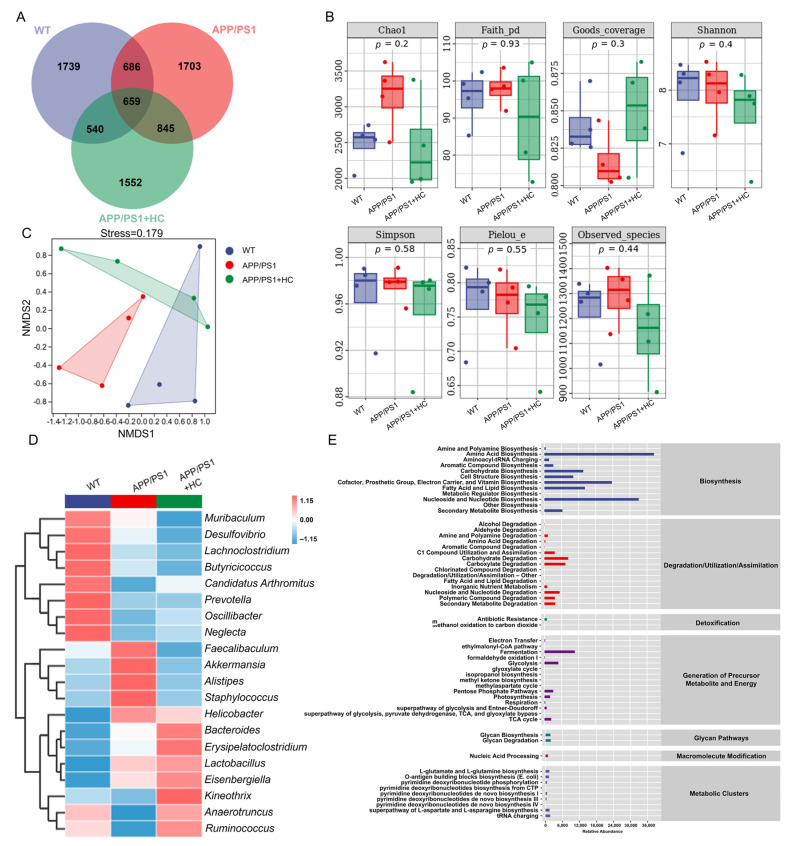
HC regulates gut microbiotain APP/PS1 mice. (**A**) Venn diagram. (**B**) α diversity was analyzed via Chao1; Faith’s phylogenetic diversity; Good’s coverage index; Shannon; Simpson; Pielou’s evenness; observed species (*n* = 4). (**C**) NMDS for unweighted UniFrac distance and β-diversity analysis. (**D**) Heatmap of the top 20 genera with average abundance obtained by UPGMA clustering based on Euclidean distance of species composition data. (**E**) The predicted abundances of secondary functional pathways based on the MetaCyc metabolic database, where the horizontal coordinate is the abundance of functional pathways and the vertical coordinate is the functional pathways of MetaCyc’s second classification level.

Mounting evidence suggests that the gut microbiota plays an essential role in the development of AD [45]. We used operational taxonomic unit (OTU) analysis to characterize differences in gut microbiota among WT mice, APP/PS1 mice, and HC-treated APP/PS1 mice. While 1739, 1703, and 1552 specific OTUs were observed in WT, APP/PS1, and HC-treated APP/PS1 mice, respectively, no significant differences were observed among them (Figure 3A). Similarly, no group differences were shown in α diversity (Figure 3B). β-diversity was analyzed using non-metric multidimensional scaling, which revealed differences in the composition of microbial communities between different groups. β-diversity analysis showed that the APP/PS1 group was more distant from the other two groups, and HC-treated APP/PS1 mice had a similar microbial composition to the WT group, highlighting the ability of HC to regulate the structure of the gut microbiota (Figure 3C). The risk of AD may be increased or decreased by gut microbial metabolites and their influence on host neurochemical alterations. Infection with pathogenic microorganisms increases the risk of developing AD [46]. A heatmap was used to analyze the top 20 flora with average abundance at the genus level, and the results showed that, compared with WT mice, APP/PS1 mice showed a decreased abundance of *Candidatus Arthromitus*, *Oscillibacter*, *Neglecta*, *Kineothrix*, *Anaerotruncus*, and *Ruminococcus*, and an increased abundance of *Faecalibaculum*, *Akkermansia*, *Alistipes*, *Staphylococcus*, and *Helicobacter*, among which *Anaerotruncus*, *Ruminococcus,* and *Alistipes* are related to oxidative stress [47,48,49], whereas HC reversed this trend (Figure 3D and Table S2). The MetaCyc database was used to detect the metabolic pathways among all groups: fermentation, glycolysis, pentose phosphate pathways, and the TCA cycle, which are implicated in oxidative stress [50] (Figure 3E). These results suggest that HC may modulate oxidative stress-mediated AD by improving the gut microbial composition and its metabolites.

### 3.5. HC Regulated the Levels of Oxidative Stress-Related Metabolites in Serum

**Figure 4:**
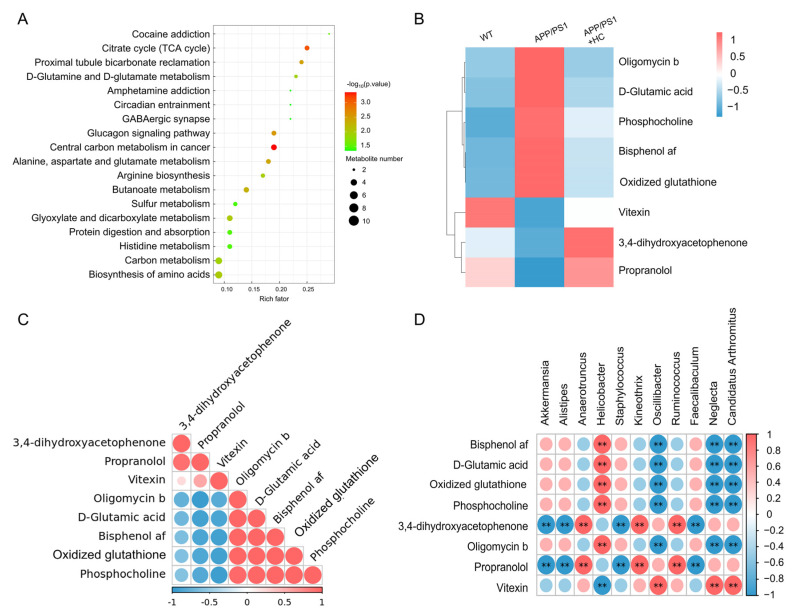
HC regulates serum metabolites in APP/PS1 mice. (**A**) KEGG enrichment pathway diagram (The deeper the bubble color, the smaller the *p* value and the more significant the enrichment degree. Bubble size is positively associated with the influencing elements) (*n* = 4). (**B**) Heatmap of eight significantly altered metabolites in HC-treated APP/PS1 mice. (**C**) Correlation coefficient diagram of serum metabolites after HC treatment. (**D**) Correlation analysis between gut microbiota and serum metabolites ** *p* < 0.01.

To investigate the neuroprotective mechanism of HC, serum metabolism was analyzed using UPLC–MS/MS. In the non-targeted serum metabolites, a total of 1035 metabolites were detected, including lipids and lipid-like molecules and organic acids and derivatives (Figure S2A); inter-group differences were evaluated via OPLS-DA analysis (Figure S2B). In the Venn diagram analysis, after HC treatment, 67 metabolites differed from those in APP/PS1 mice (Figure S2C). According to the metabolic pathway database, HC altered 18 metabolic pathways, including D-glutamine and D-glutamate metabolism, GABAergic synapses, and sulfur metabolism (Figure 4A). Compared to those in WT mice, APP/PS1 mice had decreased levels of three metabolites and increased levels of five metabolites. After HC treatment, this phenomenon was reversed (Figure 4B and Table S3). Among these metabolites, D-glutamic acid was positively correlated with oxidized glutathione levels (Figure 4C). According to the correlation analysis of the top 20 most abundant intestinal bacteria with eight kinds of altered metabolites, D-glutamic acid and oxidized glutathione levels were positively correlated with the abundance of *Helicobacter*, and negatively correlated with *C. Arthromitus* (Figure 4D), which are associated with oxidative stress.

### 3.6. Anti-Oxidative Stress Effects of HC Treatment Were Mediated by the Nrf2 Pathway

**Figure 5:**
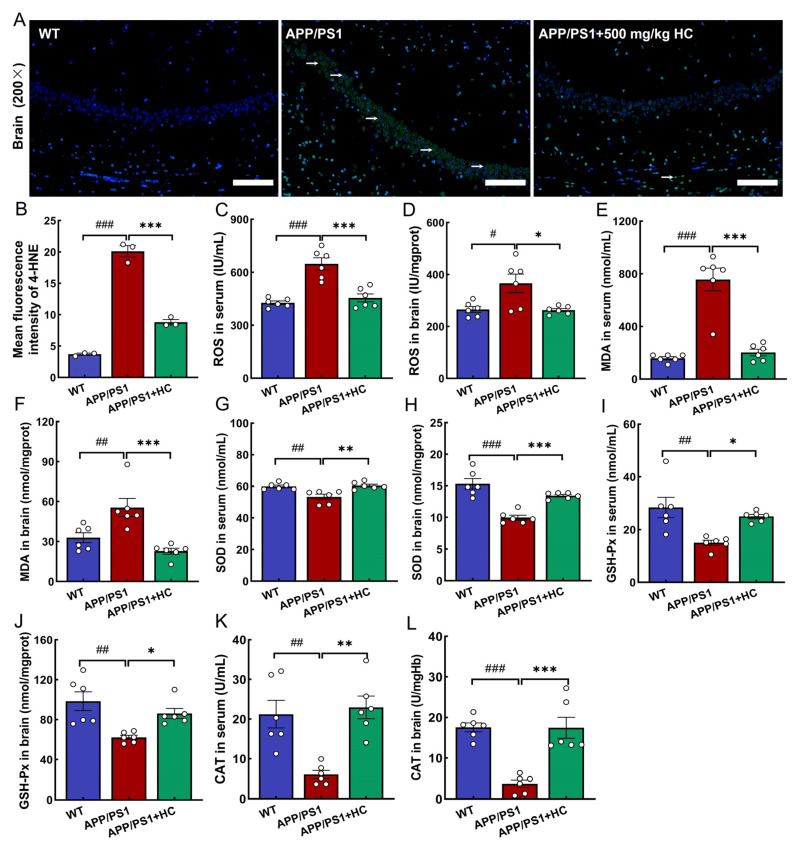
HC increased the antioxidant capacity in APP/PS1 mice. (**A**) HC reduced the expression of 4-HNE in the brain (white arrow). (**B**) Semi-quantitative data of 4-HNE. Scale bar = 100 μm for 200× magnification. HC decreased the levels of (**C**,**D**) ROS and (**E**,**F**) MDA and increased the levels of (**G**,**H**) SOD, (**I**,**J**) GSH-Px, and (**K**,**L**) CAT in serum and brain. Data are expressed as mean ± S.E.M. (*n* = 3 for (**A**,**B**), *n* = 6 for (**C**–**L**)). ^#^ *p* < 0.05, ^##^ *p* < 0.01, ^###^ *p* < 0.001 vs. WT mice; * *p* < 0.05, ** *p* < 0.01, *** *p* < 0.001 vs. APP/PS1 mice.

Oxidative stress is the excessive production of free radicals after the body is stimulated, and the antioxidant effect in the body is abnormal, which promotes the deposition of Aβ, excessive p-tau, and the apoptosis of neurons [51]. Excessive ROS causes lipid peroxidation, generates 4-HNE and MDA, and aggravates AD [52]. The body’s defense system of antioxidant enzymes reduces oxidative stress by reducing ROS. SOD, GSH-Px, and CAT are antioxidant enzymes that contribute significantly to reducing oxidative stress in vivo [53]. Compared with that in APP/PS1 mice, HC reduced the expression of 4-HNE in the brain (*p* < 0.001) (Figure 5A,B), inhibited the levels of ROS and MDA in serum and brain tissue (*p* < 0.05) (Figure 5C–F), and increased the levels of SOD (*p* < 0.01) (Figure 5G,H), GSH-Px (*p* < 0.05) (Figure 5I,J), and CAT (*p* < 0.01) (Figure 5K,L) in serum and brain tissue.

**Figure 6:**
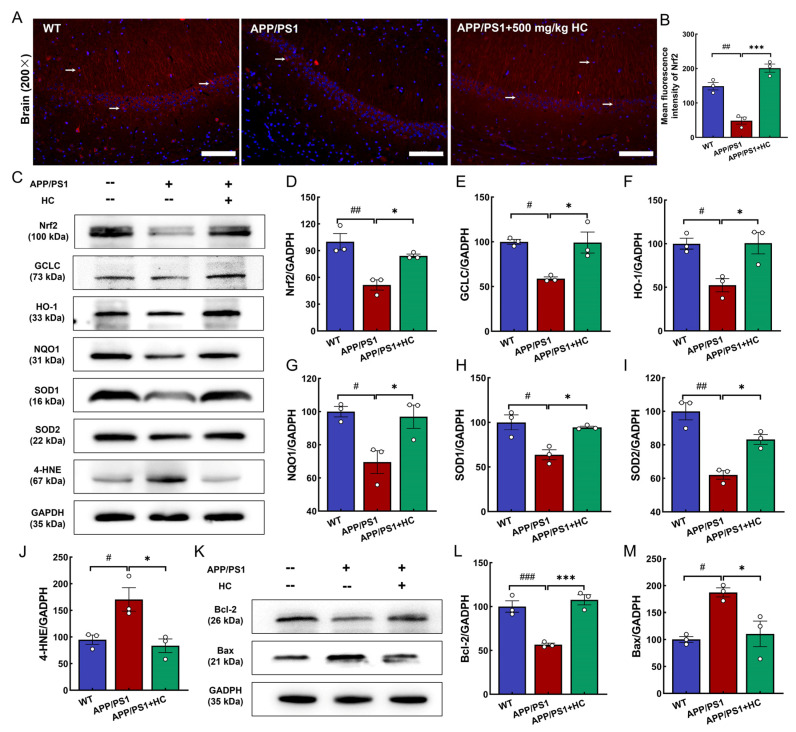
HC treatment activates the Nrf2 pathway in APP/PS1 mice. (**A**) HC increased the expression of Nrf2 in the brain (white arrow). (**B**) Semi-quantitative data of Nrf2. (**C**) HC treatment regulated Nrf2 signaling and 4-HNE expression. Quantification of the mean density of (**D**) Nrf2, (**E**) GCLC, (**F**) HO-1, (**G**) NQO1, (**H**) SOD1, (**I**) SOD2, and (**J**) 4-HNE. (**K**) HC ameliorated the concentrations of Bcl-2 and Bax. Quantification of the mean density of (**L**) Bcl-2 and (**M**) Bax. Quantitative data were normalized by GAPDH and expressed as a percentage of the corresponding WT mice. Scale bar = 100 μm for 200× magnification. Data are expressed as mean ± S.E.M. (*n* = 3). ^#^ *p* < 0.05, ^##^ *p* < 0.01, ^###^ *p* < 0.001 vs. WT mice; * *p* < 0.05, *** *p* < 0.001 vs. APP/PS1 mice.

In AD, the Nrf2 signaling pathway protects the body from oxidative stress. HC treatment upregulated the expression of Nrf2 in the brain of APP/PS1 mice (*p* < 0.001) (Figure 6A,B). To further verify the immunofluorescence results, Western blotting was used to evaluate the expression of Nrf2 and its downstream targets (HO-1, SOD1, SOD2, glyceraldehyde-3-phosphate dehydrogenase (GCLC), and NQO1) in the mouse brain. Nrf2, GCLC, HO-1, NQO1, SOD1, and SOD2 levels were significantly downregulated in APP/PS1 mice compared with those in WT mice (*p* < 0.05). After 7 weeks of HC treatment, these phenomena were strongly reversed (*p* < 0.05) (Figure 6C–I). Additionally, the expression of the oxidative damage marker 4-HNE was decreased in HC-treated APP/PS1 mice (*p* < 0.05) (Figure 6C,J). Excessive ROS damages mitochondrial DNA and induces mitochondrial dysfunction. Mitochondrial disorders were significantly influenced by proteins from the B cell lymphoma-2 (Bcl-2) family; HC increased the expression of Bcl-2 (*p* < 0.001) and reduced that of Bcl2-associated X (Bax) (*p* < 0.05) (Figure 6K–M). Above results suggesting that HC regulates Nrf2 signaling.

## 4. Discussion

In this study, the composition of HC was determined, and it was found that HC contained a variety of nutrients, among which the total sugar content was the highest. Various nutrients in HC may be involved in regulating neurodegenerative diseases. First, we identified improved memory and cognitive effects of HC in APP/PS1 mice. Second, we analyzed the neuroprotective effects of HC in APP/PS1 mice based on gut microbiome and non-targeted serum metabolomics. HC administration reversed the increase in *Helicobacter* abundance and oxidized glutathione levels in APP/PS1 mice. Finally, HC alleviated oxidative stress; reduced ROS and MDA levels; increased GSH-Px, CAT, and SOD levels; and activated the Nrf2 pathway to ameliorate AD symptoms. These findings demonstrate that HC inhibits AD progression by modulating oxidative stress, highlighting its potential as a therapeutic agent.

Currently, no medication can prevent, treat, or reverse the progression of AD [54]. Owing to the complexity of AD pathogenesis, the efficacy of single-target drug therapy is limited and has side effects [55]. Mushrooms contain various bioactive ingredients and are considered potential drug candidates owing to their safety and neuroprotective effects [56]. In the present study, HC were rich in nutrients, which improved the pathological features of AD. Extracellular Aβ deposits form plaques and accelerate tau hyperphosphorylation, thereby accelerating AD progression [57,58]. The interaction between Aβ and p-tau leads to neuronal loss and synaptic damage, resulting in cognitive decline, while plaque deposition and neurofibrillary tangles lead to direct behavioral changes in patients with AD [57]. After HC treatment, the short-term and spatial learning and memory of APP/PS1 mice were improved, the deposition of Aβ was reduced, and the expression of p-tau was downregulated, which is similar to previous findings on *H. erinaceus* [59,60], suggesting a potential therapeutic role for HC in APP/PS1 mice. Disruption of the gut microbiota induces Aβ deposition and oxidative stress, leading to memory and cognitive impairments [61]. The regulation of the gut microbiota improves AD-like symptoms. Gut microbiota a improve AD symptoms by secreting neurotransmitters and other beneficial metabolites (such as SCFAs) [62]. In APP/PS1 mice, HC increased SCFA concentrations by increasing the abundance of *Anaerotruncus* and *Ruminococcus* [47,48]. SCFAs directly cross the blood–brain barrier and act on the central nervous system to regulate redox enzymes, activate the Nrf2 pathway, and maintain redox homeostasis under physiological conditions, thereby preventing Aβ accumulation and ameliorating cognitive impairment [11,63]. In addition, SCFAs interfere with the protein–protein interactions of Aβ peptides and inhibit the formation of neurotoxic aggregates from Aβ oligomers [64]. *Alistipes* is a common bacterium associated with oxidative stress, and the abundance of *Alistipes* and *Helicobacter* are positively correlated with ROS and negatively correlated with SOD and glutathione (GSH) [49]. *Helicobacter* induces Aβ overexpression in the rat hippocampus and cortex [12]. In this study, the abundance of *Akkermansia*, *Alistipes*, and *Helicobacter* increased; meanwhile, *Anaerotruncus* and *Ruminococcus* decreased in APP/PS1 mice, which is consistent with previous studies. After HC treatment, its abundance significantly decreased, indicating a neuroprotective effect. These results suggest that HC exerts an anti-AD effect by regulating the gut microbiota, which is probably related to oxidative stress.

Serum metabolome and pathway enrichment analyses showed that HC intervention decreased the levels of D-glutamic acid and oxidized glutathione, and affected D-glutamine and D-glutamic acid metabolism in APP/PS1 mice. GSH, which interacts with ROS, plays a crucial role in the antioxidant defense system and maintenance of neuronal redox homeostasis [65]. The reduced GSH/oxidized glutathione ratio is widely used as an important index of cellular redox balance [66]. D-glutamic acid is a potent natural inhibitor of GSH synthesis, and excess glutamate inhibits the antioxidant state, induces oxidative stress, and leads to neuronal cell death [67,68]. In addition, lipid peroxidation products lead to the loss of glutamate transporter function [69]. As expected, HC reduced free radicals and lipid peroxidation and maintained cell redox homeostasis.

Based on the gut microbial and metabolomic analyses, we conjecture that the neuroprotective effect of HC on APP/PS1 mice may be related to the modulation of oxidative stress. In AD pathology, Aβ and p-tau promote the production of ROS [70], and the excessive accumulation of ROS attacks biofilms, induces lipid peroxidation, and produces 4-HNE and MDA [71]. Nrf2 enhances cellular defenses and ameliorates oxidative damage by regulating oxidative stress [72]. Many natural products have been demonstrated to act as Nrf2 inducers to prevent Aβ accumulation and tau phosphorylation by regulating oxidative stress, thereby improving the physiological function of AD mice. When stimulated by oxidative stress, Nrf2 induces the expression of downstream regulatory genes such as HO-1, NQO1, and GCLC, and activates antioxidant enzymes (SOD and GSH-Px) [15,73,74], whose defense mechanisms improve oxidative stress. SOD is the first line of defense against free radicals and converts superoxide anions into H_2_O_2_, which is catalyzed by GSH-Px and CAT to decompose into oxygen and water, thus acting as a protective agent against ROS toxicity [75,76]. In APP/PS1 mice, HC not only upregulated the levels of the antioxidant enzymes SOD, GSH-Px, and CAT in brain tissue and serum, but also downregulated the expression of 4-HNE and MDA in the brain. The expression of Nrf2 and its driving genes, such as NQO1, HO-1, and GCLC, were decreased in AD brains [15]. In this study, HC reduced the production of lipid peroxides and increased the levels of antioxidant enzymes by activating the Nrf2 signaling and regulating gut microbiota, thus preventing oxidative damage and delaying AD progression.

## 5. Conclusions

In conclusion, we confirmed that HC alleviated oxidative stress and cognitive impairment in APP/PS1 mice by activating Nrf2 signaling and regulating the gut microbiota, thus playing an anti-AD role and suggesting that HC is a promising potential treatment for AD. This study had some limitations. Although the composition of HC has been determined, the active ingredient with neuroprotective effects has not been confirmed, and further research is needed.

## Figures and Tables

**Table 1:** Main composition of HC.

	Compounds	Contents (%)	Compounds	Contents (%)
General nutritional composition	Total sugar	33.40	Total triterpenoids	3.32
	Crude protein	16.00	Total saponins	1.10
	Total ash	11.70	Total flavonoids	0.88
	Reducing sugar	7.95	Total alkaloids	0.57
	Crude fat	6.30	Total sterol	0.43
	Crude fiber	4.60	Total phenol	0.18
	Compounds	Contents (mg/kg)	Compounds	Contents (mg/kg)
Minerals	K	4.02 × 10^4^	Zn	64.60
	Mg	1.09 × 10^3^	Fe	50.90
	Ca	280.00	Mn	7.97
	Na	110.00	Se	0.04
Heavy metals	Cu	113.00	As	0.11
	Cr	0.76	Pb	0.04
	Cd	0.20	Hg	0.01

Above results are shown to retain two decimal places.

## Data Availability

Not applicable.

## Supplementary Materials

The following supporting information can be downloaded at: https://www.mdpi.com/article/10.3390/nu15173799/s1, Table S1: Details of antibodies used in Western blotting; Table S2: Relative abundance of top 20 genera among WT, APP/PS1, and APP/PS1+HC; Table S3: The differential metabolites of serum among WT, APP/PS1, and APP/PS1+HC; Figure S1: Safety evaluation of APP/PS1 mice treated with HC; Figure S2: Classification, quantity, and multidimensional statistical analysis of metabolites.
