# Supplementary material for: Hericium coralloides Ameliorates Alzheimer’s Disease Pathologies and Cognitive Disorders by Activating Nrf2 Signaling and Regulating Gut Microbiota
Source: Nutrients. 2023 Aug 30;15(17):3799. doi: 10.3390/nu15173799 (PMC10489620; doi:10.3390/nu15173799)
Supplement: Supplementary file 1 [file nutrients-15-03799-s001.zip › nutrients-2557593-supplementary.pdf]

Supplementary Data

Table S1. Details of antibodies used in Western blotting.

Antibody	Molecular weight	Catalog number	Dilution	Company	Area
t-tau	79 kDa	A0002	1:2000	ABclonal	Wuhan, China
p-tau	79 kDa	ab32057	1:2000	Abcam	Cambridge, MA, USA
$A\beta_{1-40}$	4.3 kDa	bs-0877R	1:2000	Bioss	Beijing, China
$A\beta_{1-42}$	4.4 kDa	bs-0107R	1:2000	Bioss	Beijing, China
GAPDH	35 kDa	E-AB-40337	1:2000	Elabscience	Wuhan, China
Nrf2	100 kDa	A1244	1:1000	ABclonal	Wuhan, China
GCLC	73 kDa	bs-23393R	1:1500	Bioss	Beijing, China
HO-1	33 kDa	AF5393	1:1000	Affinity	Cincinnati, OH, USA
NQO1	31 kDa	A19586	1:2000	ABclonal	Wuhan, China
SOD1	16 kDa	A12537	1:1000	ABclonal	Wuhan, China
SOD2	22 kDa	A1340	1:1000	ABclonal	Wuhan, China
4-HNE	67 kDa	bs-6313R	1:1000	Bioss	Beijing, China
Bcl-2	26 kDa	A0208	1:1000	ABclonal	Wuhan, China
Bax	21 kDa	A19684	1:1000	ABclonal	Wuhan, China
goat anti-mouse		E-AB-1001	1:4000	Elabscience	Wuhan, China
goat anti-rabbit		E-AB-1003	1:4000	Elabscience	Wuhan, China

Table S2. Relative abundance of top 20 genera among WT, APP/PS1 and APP/PS1+HC.

Taxa	WT	APP/PS1	APP/PS1+HC
Muribaculum	0.168931	0.147624	0.119309745
Lactobacillus	0.024037	0.090404	0.106769995
Lachnoclostridium	0.069882	0.04285	0.035904689
Eisenbergiella	0.012297	0.025235	0.031373288
Akkermansia	0.006255	0.051867	0.000128735
Desulfovibrio	0.023127	0.011201	0.003374753
Bacteroides	0.004681	0.012425	0.019031598
Alistipes	0.008606	0.01562	0.008180541
Erysipelatoclostridium	0.00457	0.009511	0.016109862
Anaerotruncus	0.009517	0.005499	0.010060246
Helicobacter	0.00188	0.012341	0.009718913
Staphylococcus	0.002836	0.009946	0.002749092
Kineothrix	0.003342	0.002538	0.00727808
Butyricicoccus	0.004694	0.002369	0.001389474
Oscillibacter	0.00455	0.001589	0.002256994

Ruminococcus	0.002902	0.001924	0.00329547
Prevotella	0.005757	0.000174	0.00004539
Faecalibaculum	0.001701	0.004107	0.00004997
Neglecta	0.003233	0.001031	0.001406
Candidatus Arthromitus	0.003019	0.000441	0.001529127

Data are presented as the mean.

Table S3. The differential metabolites of serum among WT, APP/PS1 and APP/PS1+HC.

**Metabolites**	**WT**	**APP/PS1**	**APP/PS1+HC**	**ANOVA *P* value**
Phosphocholine	9334151.018	26085118.25	13626510.64	0.001338
Oligomycin b	2080200.549	6223581.442	2123645.369	0.01381
Bisphenol af	145204716.6	241463850	164955740.4	0.020436
3,4-dihydroxyacetophenone	4243171.542	3899588.444	4898134.431	0.015564
Vitexin	575009.3181	288833.2647	404925.8763	0.026925
Propranolol	1857670.674	821794.0851	2385704.623	0.04404
D-Glutamic acid	54131865.59	67947340.77	55742368.18	0.003671
Oxidized glutathione	15201929.28	18239389.14	15857783.28	0.015878

Data are presented as the mean.

Differences are considered statistically significant at ANOVA *P* value <0.05

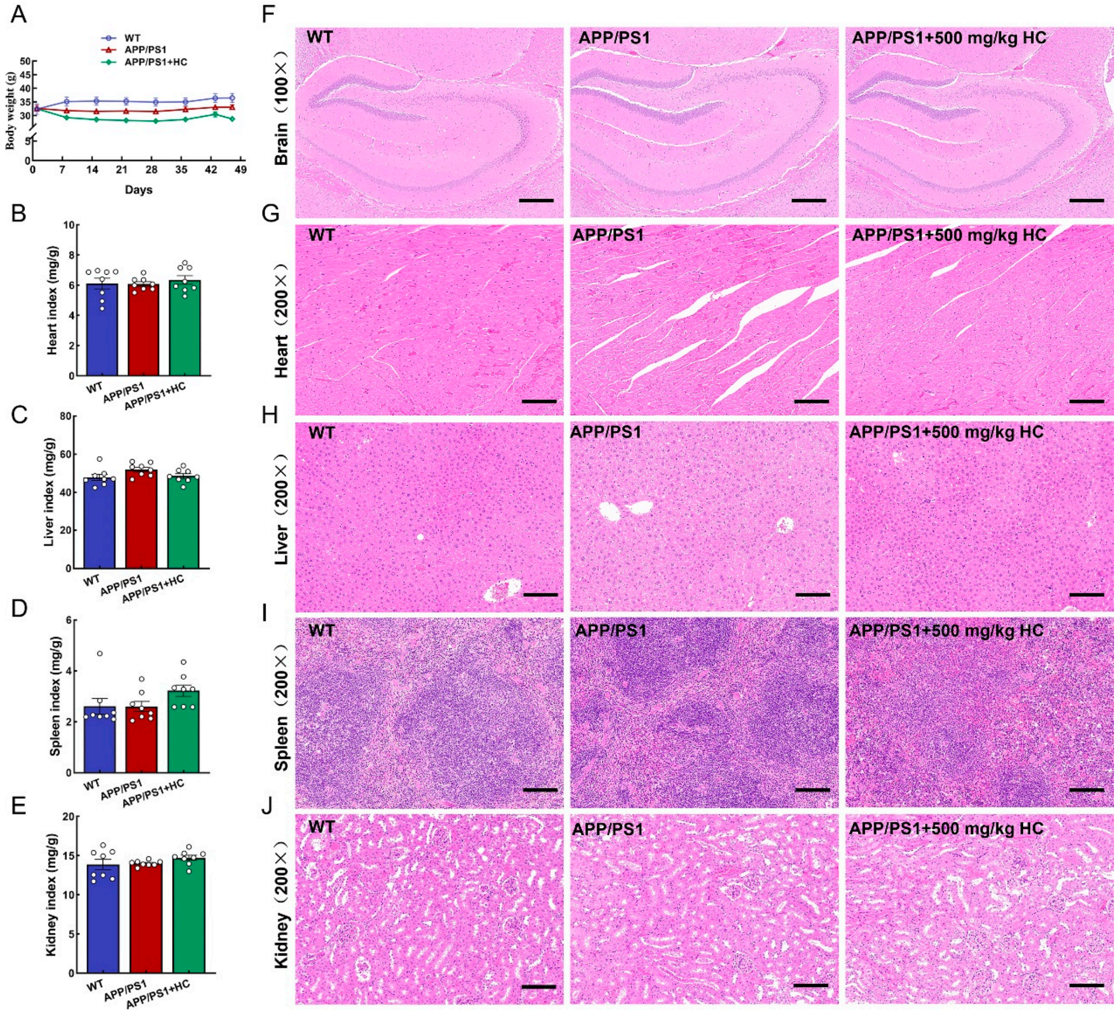

Figure S1 Safety evaluation of APP/PS1 mice treated with HC. (**A**) The body weight changes (n = 8). (**B**) Heart index, (**C**) liver index, (**D**) spleen index, and (**E**) kidney index of APP/PS1 mice (n = 8). Representative images of H&E staining of the (**F**) brain, (**G**) heart, (**H**) liver, (**I**) spleen, and (**J**) kidney (scale bar: 100 μm) (n = 3). Data are expressed as mean ± S.E.M..

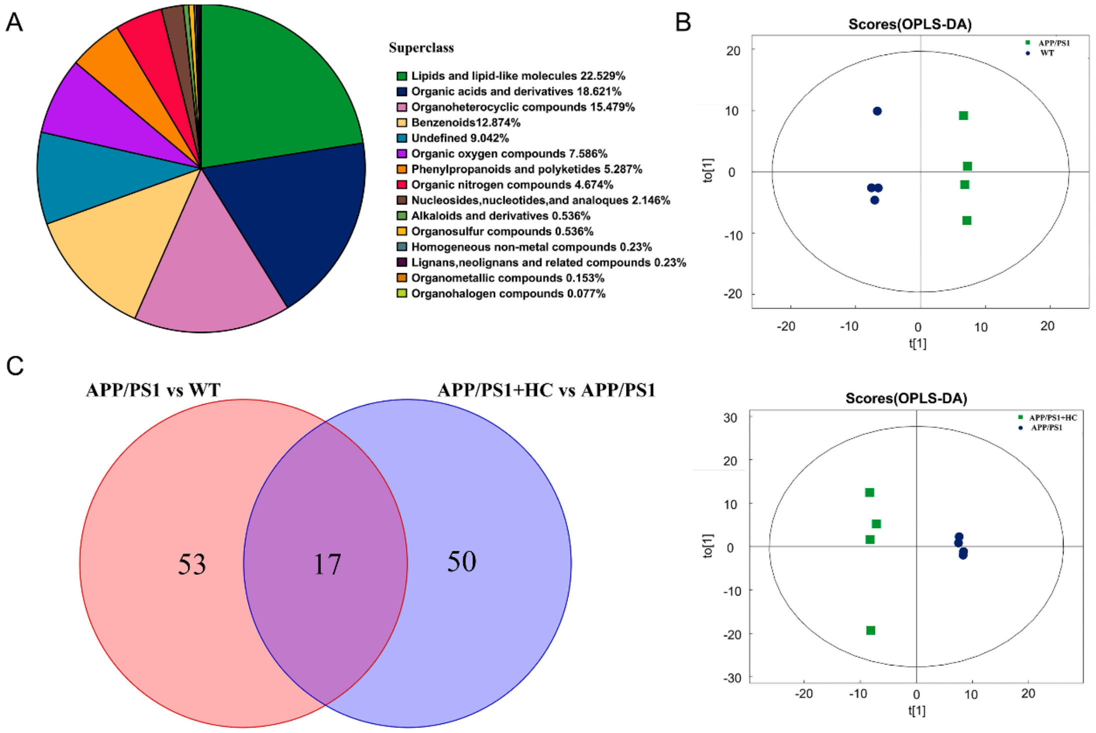

Figure S2 Classification, quantity and multidimensional statistical analysis of metabolites. (**A**) The proportion of identified metabolites in each chemical classification. (**B**) OPLS-DA score map of the WT mice and APP/PS1 mice (above), OPLS-DA score map of HC treated APP/PS1 mice and APP/PS1 mice (below) (*n* = 4). (**C**) Venn diagram.
